# A scoring system combining clinical, radiological, and histopathological examinations for differential diagnosis between lipoma and atypical lipomatous tumor/well-differentiated liposarcoma

**DOI:** 10.1038/s41598-021-04004-1

**Published:** 2022-01-07

**Authors:** Yohei Asano, Shinji Miwa, Norio Yamamoto, Katsuhiro Hayashi, Akihiko Takeuchi, Kentaro Igarashi, Hirotaka Yonezawa, Yoshihiro Araki, Sei Morinaga, Takayuki Nojima, Hiroko Ikeda, Hiroyuki Tsuchiya

**Affiliations:** 1 Department of Orthopaedic Surgery, Kanazawa University Graduate School of Medical Sciences, Kanazawa University, 13–1 Takaramachi, Kanazawa city, Ishikawa 920–8641 Japan; 2 Department of Pathology, Kanazawa University Hospital, 13–1 Takaramachi, Kanazawa city, Ishikawa 920–8641 Japan

**Keywords:** Oncology, Signs and symptoms

## Abstract

This study evaluated the diagnostic accuracy of clinical, radiological, and histopathological examinations for differential diagnosis between atypical lipomatous tumor (ALT)/well-differentiated liposarcoma (WDLS) and lipoma, and aimed to develop a new combined scoring system for the preoperative diagnosis of ALT/WDLS. Eighty-nine lipomas and 56 ALT/WDLS were included and their clinical characteristics, magnetic resonance imaging (MRI) findings, histological findings by hematoxylin and eosin (HE) staining were investigated. Then, univariate and multivariate logistic regression analyses were performed for the findings, and a combined scoring system consisted of predictive factors of ALT/WDLS was developed. The univariate and multivariate logistic regression analyses revealed that tumor location (lower extremity), deep site, size (> 11 cm), thick septa (> 2 mm), enhancement of septa or nodular lesions, and lipoblasts were significantly different for the diagnosis of ALT/WDLS. We developed a combined scoring system based on the six predictive factors (total 0–16 points, the cutoff was 9 points). The area under the curve was 0.945, and sensitivity was 87.6% and specificity was 91.1% by the receiver operating characteristics curve. This combined scoring system does not require special equipment and reagents such as fluorescence in situ hybridization (FISH), and anyone can use it easily in many medical institutions with high diagnostic accuracy.

## Introduction

Adipocytic tumors are the most common soft tissue tumors, and the frequency of lipoma and atypical lipomatous tumor (ALT)/well-differentiated liposarcoma (WDLS) is high in clinical practice^1^. Most of the adipocytic tumors are either lipomas or ALTs, accounting for 40%–45% of all tumors of the adipose tissue^2,3^. On the other hand, liposarcomas are the most common soft tissue sarcoma^4^. ALT/WDLS is the most common subtype of liposarcoma, accounting for 40%–45% of all liposarcomas^5,6^. It has been reported that ALT and WDLS are essentially synonymous, as the lesions are morphologically and karyotypically identical distinguished only by the tumor site. Adipocytic tumors located in the retroperitoneal or regions in which the tumor cannot be resected with a sufficient margin are termed WDLS^2^, whereas those located in the extremities and superficial sites are classified as ALT^1^.

It has been difficult to differentiate between ALT/WDLS and lipoma preoperatively because their magnetic resonance imaging (MRI) findings are similar^1^, and small biopsy samples are insufficient for histopathological diagnosis^7^. The selection of surgical resection margins in these tumors remains controversial. ALT and WDLS are classified as intermediate (locally aggressive) and malignant tumors, respectively, and they have a high risk of local recurrence than lipoma^7^. Additionally, as they may undergo dedifferentiation and become malignant tumors^8^, some patients need wider resection margins while still avoiding dysfunction. Therefore, the preoperative differential diagnosis between ALT/WDLS and lipoma is critical for the determination of the resection margin.

Although there have been some reports regarding the usefulness and high accuracy of the assessment, such as fluorescence in situ hybridization (FISH) for murine double-minute 2 (*MDM2*) and cyclin-dependent kinase 4 (*CDK4*) for the diagnosis of ALT/WDLS^9–16^, these technologies require special equipment and reagents, so not all medical institutions can perform this examination^7^. The assessment with MRI and hematoxylin and eosin (HE) staining remains the standard method of diagnosis for adipocytic tumors, so it is crucial to determine the diagnosis by a comprehensive assessment of clinical, radiological, and histopathological examinations. Here, we evaluated the diagnostic accuracy of various findings in some examinations and aimed to develop a combined scoring system based on the accuracy of each finding for the preoperative differential diagnosis between ALT/WDLS and lipoma.

## Results

The 145 participants of the development cohort comprised 77 men and 68 women, with a mean age of 58.8 ± 14.7 years (range 18–87 years). Based on the diagnoses established by the pathologists, the numbers of patients in the ALT/WDLS and lipoma groups were 56 and 89, respectively (Table 1). Of the 56 cases of ALT/WDLS, 46 cases underwent FISH for *MDM2* and 37 cases (80.4%) were positive and diagnosed definitively. The other 10 cases were diagnosed based on histological features^2,17^ or positive of FISH for *CDK4*. All ALT/WDLS cases were followed up after surgery and recurrence was observed in 2 cases. Clinical findings showed that the mean age of patients in the ALT/WDLS group was significantly higher than that in the lipoma group (64.0 ± 13.3 vs. 55.7 ± 14.6, p < 0.001). All tumors in the ALT/WDLS group were located deep (100% vs. 67.4%, *p* < 0.0001) and significantly larger than those in the lipoma group (17.2 ± 6.7 cm vs. 9.7 ± 4.2 cm, *p* < 0.0001) (Table 1). On MRI, every criterion showed a significant difference between the ALT/WDLS and lipoma groups (thick septa (> 2 mm); *p* < 0.0001; enhancement; *p* < 0.0001; and neurovascular involvement, *p* = 0.0225). The histological findings by HE staining, such as nuclear atypia and enlargement, difference in size of adipocytes and lipoblasts, and FISH examination for *MDM2* and/or *CDK4* showed a significant differences between them (*p* < 0.0001). Only the proliferation of fibrous septa has a non-significant p-value of 0.949 (Table 1).

**Table 1:** Characteristics of patients, MRI, and histopathological findings.

	ALT/WDLS (N = 56)	Lipoma (N = 89)	*p* value
**Clinical findings**			
Age			< 0.001
Median (range)	64.0 ± 13.29 (30–87)	55.7 ± 14.60 (18–83)	
Sex: n (%)			0.547
Male	32 (57.1%)	45 (50.6%)	
Female	24 (42.9%)	44 (49.4%)	
Symptoms: n (%)	6 (10.7%)	4 (4.5%)	0.150
Tumor site: n (%)			< 0.0001
Upper limb	5 (8.9%)	29 (32.6%)	
Lower limb	39 (69.7%)	24 (27.0%)	
Other	12 (21.4%)^a^	36 (40.4%)^b^	
Tumor depth: n (%)			< 0.0001
Superficial	0 (0%)	29 (32.6%)	
Deep	56 (100%)	60 (67.4%)	
Tumor size (cm)			< 0.0001
Median	17.2 ± 6.7	9.7 ± 4.2	
**MRI findings: n (%)**			
Thick septa (> 2 mm)	52 (92.9%)	52 (58.4%)	< 0.0001
Enhancement (septa and/or nodular lesions) (n = 20)	20 (100%)	4 (20.0%)	< 0.0001
Neurovascular involvement	5 (8.9%)	1 (1.1%)	0.023
**Histopathological findings: n (%)**			
Nuclear atypia	10 (17.9%)	0 (0%)	< 0.0001
Nuclear enlargement	26 (46.4%)	8 (9.0%)	< 0.0001
Difference in size of adipocytes	26 (46.4%)	24 (27.0%)	0.016
Proliferation of fibrous septa	2 (3.6%)	3 (3.4%)	0.949
Lipoblasts	11 (19.6%)	1 (1.1%)	< 0.0001
**FISH: positive number / n = total number (%)**			
MDM2	37/n = 46 (80.4%)	0/n = 73 (0%)	< 0.0001
CDK4	18/n = 33 (54.5%)	0/n = 49 (0%)	< 0.0001

^a^neck (n = 4), chest (n = 2), retroperitoneum (n = 2), and back (n = 2).

^b^neck (n = 15), chest (n = 13), back (n = 6), and abdomen (n = 2).

*MRI* magnetic resonance imaging, *ALT* atypical lipomatous tumor, *WDLS* well-differentiated liposarcoma, *FISH* Fluorescence in Situ Hybridization, *MDM2* murine double-minute 2, *CDK4* cyclin-dependent kinase 4.

The tumor size was analyzed using the receiver operating characteristic (ROC) curve, with the cutoff value was set to 11 cm. In previous studies, cutoff values of tumor size for differential diagnosis between lipoma and ALT/WDLS were 10–13 cm^1,18^, and our result was consistent with those studies. Univariate analyses revealed that age, tumor location, depth and size, thick septa (> 2 mm), enhancement of septa or nodular lesions, histological findings excluding the proliferation of fibrous septa, and FISH examination for *MDM2* and/or *CDK4* were significantly different (Table 2). Among these factors, the odds ratio of tumor depth, nuclear atypia, and FISH examination were extremely higher than other factors. Particularly, nuclear atypia and FISH examination were not positive in the lipoma group in this study, and the specificity of these parameters for the diagnosis of ALT/WDLS was 100%. If nuclear atypia is found in the specimens of the adipocytic tumor, the diagnosis of lipoma is excluded. *MDM2* and *CDK4* gene amplification by FISH examination is considered the gold standard for the differential diagnosis between ALT/WDLS and lipoma^15^, and these gene amplifications are not observed in lipoma. From these results, nuclear atypia and FISH examination, which are used for the definitive diagnosis of ALT/WDLS, were excluded in this study. We aimed to develop a scoring system in which these factors were negative.

**Table 2:** Univariate and multivariate logistic regression analysis of predictive factors for ALT/WDLS.

	Univariate analysis		Multivariate analysis		
	Odds ratio (95% CI)	*p* value	Odds ratio (95% CI)	*p *value	Standard partial regression coefficient
**Clinical findings**					
Age	4.26 (1.87–9.74)	< 0.001	4.16 (0.93–18.60)	0.062	1.425
Sex	1.30 (0.67–2.56)	0.440			
Symptoms	2.55 (0.69–9.47)	0.162			
Tumor site					
Lower limb vs Upper limb or Other	6.21 (2.97–13.00)	< 0.0001	5.51 (1.51–20.10)	0.001	1.706
Depth	> 1000 (0.00-Inf)	< 0.0001	> 1000 (0.00-Inf)	< 0.0001	18.829
Tumor size (> 11 cm)	12.40 (5.20–29.60)	< 0.0001	4.83 (1.37–17.00)	0.014	1.574
**MRI findings**					
Thick septa (> 2 mm)	9.25 (3.08–27.80)	< 0.0001	5.56 (1.09–28.30)	0.039	1.715
Enhancement (septa or nodular lesion)	24.50 (7.90–76.10)	< 0.0001	15.10 (2.91–77.90)	0.001	2.711
Neurovascular involvement	8.63 (0.98–75.90)	0.052			
**Histopathological findings**					
Nuclear atypia	> 1000 (0.00-Inf)	< 0.0001			
Nuclear enlargement	8.77 (3.58–21.50)	< 0.0001	1.69 (0.40–7.17)	0.480	0.521
Difference in size of adipocytes	2.35 (1.16–4.74)	0.018	0.91 (0.26–3.20)	0.888	− 0.090
Proliferation of fibrous septa	1.06 (0.17–6.56)	0.949			
Lipoblasts	21.5 (2.69–172.00)	0.004	17.60 (1.08–286.00)	0.043	2.548
**FISH**					
MDM2 and/or CDK4	> 1000 (0.00-Inf)	< 0.0001			

*MRI* magnetic resonance imaging, *CI* confidence interval, *Inf* infinity, *ALT* atypical lipomatous tumor, *WDLS* well-differentiated liposarcoma, *FISH* Fluorescence in Situ Hybridization, *MDM2* murine double-minute 2, *CDK4* cyclin-dependent kinase 4.

Subsequently, multivariate analysis incorporating the variables with p < 0.05 in the univariate analysis was performed. In the analysis, tumor location (lower extremity), depth (deep site), and size (> 11 cm), thick septa (> 2 mm), enhancement of septa or nodular lesions, and lipoblasts were significantly different, and they had a high accuracy in the diagnosis of ALT/WDLS (Table 2).

**Figure 1:**
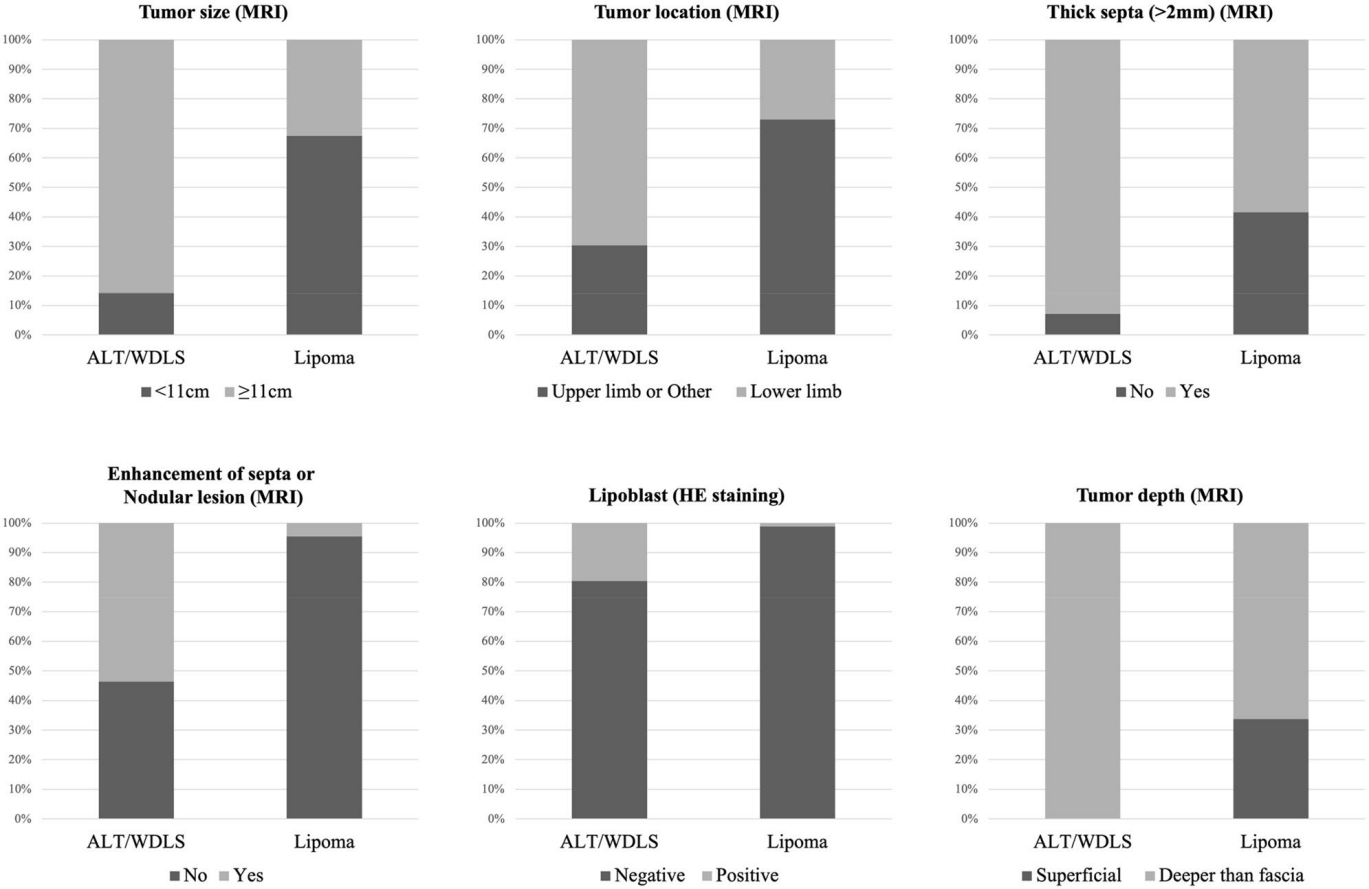
The distributions of six predictive factors of the scoring system in the ALT/WDLS and lipoma groups.

**Figure 2:**
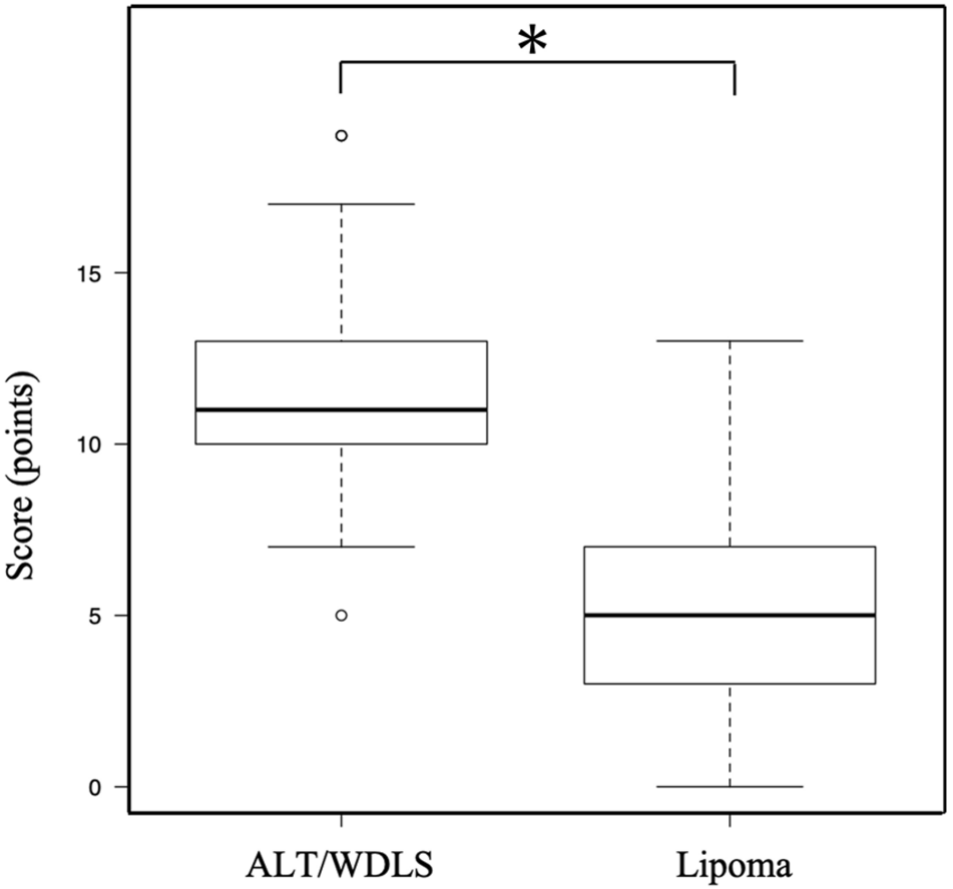
Comparison of the total score of the ALT/WDLS and lipoma groups in the development cohort (mean points were 11.9 vs. 5.6, *p < 0.0001).

**Figure 3:**
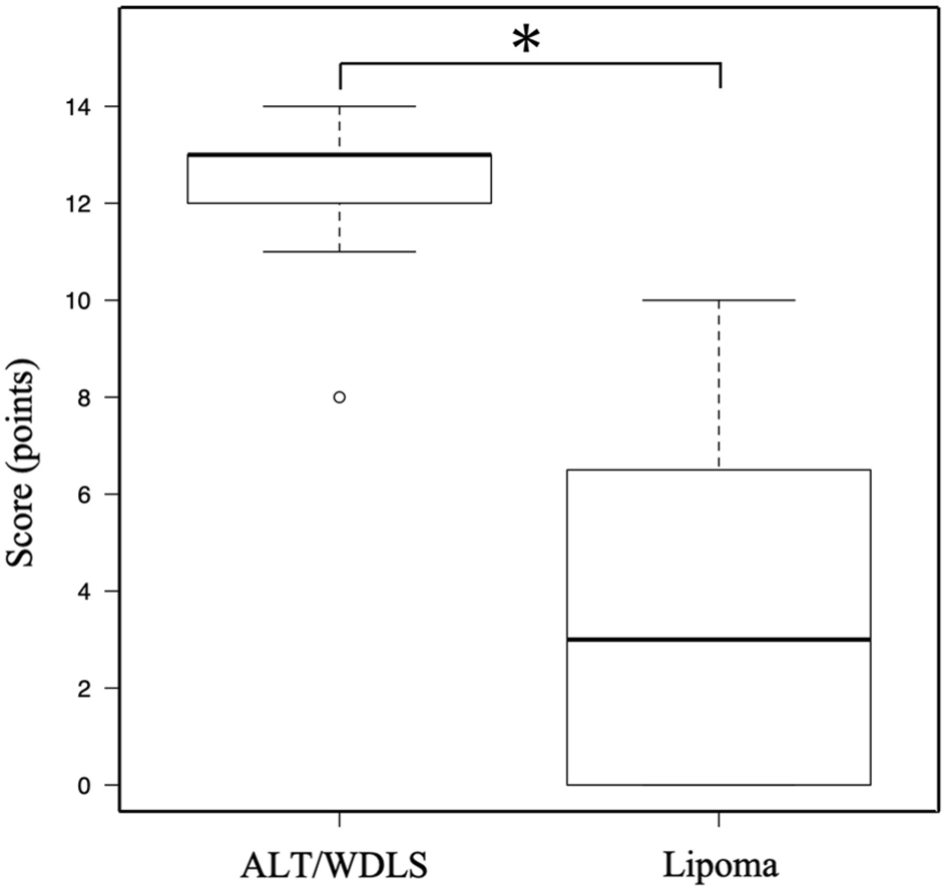
Comparison of the total score of the ALT/WDLS and lipoma groups in the validation cohort (mean points were 12.2 vs. 3.4, *p < 0.0001).

Based on these results, a combined scoring system for the preoperative differential diagnosis of ALT/WDLS was developed according to the predictive factors (Table 3). For these factors, the distributions of ALT/WDLS and lipoma groups were shown in Fig. 1. With reference to the odds ratio, the points of this score were set as follows: < 5, 1 point; 5–10, 2 points; and > 10, 3 points. The odds ratio of the tumor depth was extremely high, so the point was set to 5 points. The total points ranged from 0 to 16, and the mean number of points of the ALT/WDLS group was significantly higher than that of the lipoma group (mean points of ALT/WDLS and lipoma groups were 11.9 versus 5.6, *p* < 0.0001) (Fig. 2). The cutoff value was 9 points based on the ROC curve analysis. The increased points suggested that the possibility of ALT/WDLS was higher, with a sensitivity of 87.6% and a specificity of 91.1%. The analysis of the ROC curve also showed an area under the curve (AUC) of 0.945, which indicated that the accuracy of this combined scoring system for the differential diagnosis of ALT/WDLS was very high. The validation cohort was summarized in Table 4, and in the cross-validation, the sensitivity, specificity, NPV, PPV, diagnostic accuracy, and kappa coefficient of the combined scoring system were 80.0%, 96.7%, 93.5%, 88.9%, 92.5%, and 0.793, respectively (Table 5). The mean score of the ALT/WDLS group was significantly higher than that of the lipoma group (mean points of ALT/WDLS and lipoma groups were 12.2 versus 3.4, *p* < 0.0001) (Fig. 3). If needle biopsy could not be performed, the total points were ranged from 0 to 13, excluding lipoblast, and the ROC curve analysis showed the cutoff value was 10 points and AUC was 0.943 with a sensitivity of 94.4% and a specificity of 80.4%.

**Table 3:** A combined scoring system for the preoperative differential diagnosing of ALT/WDLS.

Predictive factors	Odds ratio	Points
Tumor size (MRI)	4.83	
≤ 11 cm		0
> 11 cm		1
Tumor location (MRI)	5.51	
Upper limb or Other*		0
Lower limb		2
Thick septa (> 2 mm) (MRI)	5.56	
No		0
Yes		2
Enhancement of septa or nodular lesion (Enhanced MRI)	15.10	
No		0
Yes		3
Lipoblast (HE staining)	17.60	
Negative		0
Positive		3
Depth (MRI)	> 1000	
Superficial		0
Deeper than fascia		5
Cutoff value		9 / 16 points

The total points ranged from 0 to 16, and the cutoff value was 9 points.

*: neck, chest, abdomen, back, and retroperitoneum.

*MRI* magnetic resonance imaging, *HE* hematoxylin and eosin, *ALT* atypical lipomatous tumor, *WDLS* well-differentiated liposarcoma.

**Table 4:** Characteristics of the validation cohort.

	Validation cohort (N = 40)
**Clinical findings**	
Age	58.3 ± 11.97 (32–80)
Sex	
Male	19
Female	21
**Tumor site**	
Upper limb	14
Lower limb	13
Other	13
Neck	6
Back	7
**Diagnosis**	
ALT/WDLS	9
Lipoma	31

*ALT* atypical lipomatous tumor, *WDLS* well-differentiated liposarcoma.

**Table 5:** The predictive powers of the combined scoring system for the differential diagnosis between ALT/WDLS and lipoma in the development and validation cohort.

	Sensitivity	Specificity	NPV	PPV	Accuracy	Kappa coefficient
Development cohort	87.6%	91.1%	87.6%	91.1%	89.0%	0.772
Validation cohort	80.0%	96.7%	93.5%	88.9%	92.5%	0.793

*NPV* negative predictive value, *PPV* positive predictive value.

## Discussion

A lipoma is a benign tumor that can usually be treated conservatively^1,7^. Even if the tumor size is large, surgical treatment is not necessary as long as it remains asymptomatic. On the other hand, ALT/WDLS is the most common subtype of liposarcoma, and the treatment for these tumors is controversial^1^ because it is reported that 1%–4% of them undergo dedifferentiation^19–21^ and may become malignant tumors^2,8,19,22^. If preoperative differential diagnosis between ALT/WDLS and lipoma is easy in ALT/WDLS patients, surgical resection is recommended before dedifferentiation^1^. The decision of the resection margin of ALT/WDLS is difficult because the local recurrence rate is high^7^. In our department, to prevent the recurrence, if the border between the normal tissue and the lipomatous tumor is ill-defined on MRI, sub-extensive resection is performed including the surrounding soft tissue, such as muscle tissue^23^. It has been reported that there was no significant difference in the recurrence rate between the wide and marginal resection in ALT^24^, and "conservative" surgery, aiming to preserve major vessels or nerves, may be recommended for deep-seated ALT/WDLS^23,25–27^. Moreover, in recent years, Vos et al. reported that observation could be a reasonable option for selected patients with extremity WDLS^28^. However, insufficient resection may increase the risk of recurrence and dedifferentiation and resulting in the need for more surgery to perform additional tissue resection. For these reasons, it is crucial to make an accurate differential diagnosis between ALT/WDLS and lipoma for the preoperative plan of the appropriate resection margin and the appropriate selection of patients who can be observed.

For the differential diagnosis, previous studies reported critical factors such as clinical findings (older age^13,29,30^, tumor size [> 10 cm]^1,12,29,31^, tumor site [lower extremity]^12,29,31^, and deep-seated location^1^) and MRI findings (thick septa [> 2 mm]^32,33^, fat content less than 75%^30^, and contrast enhancement^1^). In this study, a multivariate analysis was performed to evaluate the predictive factors for ALT/WDLS, and there was a significant difference in tumor site (lower extremity), depth, size (> 11 cm), thick septa, and enhancement of septa or nodular lesions. All of these were consistent with the previously mentioned factors, demonstrating high accuracy for the differential diagnosis of ALT/WDLS. MRI findings have high sensitivity but low specificity for the diagnosis of ALT/WDLS, and it has been reported that it may be difficult to decide the diagnosis of them by only MRI findings^7,29^. On the other hand, Brisson et al. reported that histopathological examination could be used to distinguish ALT/WDLS from lipomas^29^, so for adipocytic tumors, needle biopsies have been generally performed preoperatively to obtain the diagnosis^2^.

Histopathological findings in the multivariate analysis showed that lipoblasts were statistically significant for the diagnosis of ALT/WDLS. Traditionally, lipoblasts have been emphasized as a histopathological finding in the diagnosis of liposarcoma^34^. However, lipoblasts are also observed in benign lipogenic tumors such as spindle cell/pleomorphic lipoma. Furthermore, it has been reported that they are not always observed in ALT/WDLS^35^. Although the diagnostic accuracy of lipoblasts in the diagnosis of ALT/WDLS has not been clarified, our study revealed that their sensitivity was low (19.6%), but the specificity was very high (98.9%). Only lipoblasts cannot make a definitive diagnosis of ALT/WDLS, but they may be important factors in the diagnosis by comprehensive evaluations together with clinical and radiological examinations.

In this study, nuclear atypia had a significant difference in the differential diagnosis of ALT/WDLS by univariate analysis. ALT/WDLS features a mature adipocytic tumor showing atypical hyperchromatic nuclei^2^, which is consistent with our results. No nuclear atypia is found in lipoma, which is a benign tumor, so this characteristic is used for the rule out of the diagnosis of these tumors. The specificity of nuclear atypia was 100% in the diagnosis of ALT/WDLS in this study. However, needle biopsies may not provide enough sample for the identification of unequivocal atypical cells^7,36^, and the atypical stromal cells sometimes scatter throughout the lesion; therefore, in some cases, the difference between ALT/WDLS and lipoma may be subtle challenging the differential diagnosis process^36^.

In addition, FISH examination for *MDM2* and *CDK4* gene amplification has provided the most accuracy for the diagnosis of ALT/WDLS^9–13,15,16^, and it is considered the gold standard for the differential diagnosis between ALT/WDLS and lipoma^15^. In this study, as well as previous studies, *MDM2* and/or *CDK4* amplification by FISH examination showed a significant difference as a predictive factor for ALT/WDLS. Furthermore, the specificity was 100%. *MDM2* gene amplification in FISH examination has high sensitivity and specificity^15^, and this finding has been used for the definitive diagnosis of ALT/WDLS. However, similar to nuclear atypia, an insufficient sample and the selection of an inappropriate needle biopsy site might complicate the accurate exclusion of a diagnosis of ALT/WDLS due to the absence of *MDM2* gene amplification. Generally, HE staining in the histopathological examination can be performed in many institutions, but FISH examination requires special equipment and reagents, which not all institutions are equipped to perform^7^. For these reasons, this study excluded the nuclear atypia and FISH examination used for the definitive diagnosis of ALT/WDLS.

In fact, of the 56 cases that diagnosed ALT/WDLS by the resected specimens, 45 cases had obtained an accurate diagnosis by needle biopsy and the diagnostic accuracy was 80.3% in this study. Pohlig et al. reported that the diagnostic accuracy of needle biopsy and open biopsy for soft tissue tumors were 84.6% and 100%, respectively^37^. Although our diagnostic accuracy of needle biopsy was almost consistent with this report, it was less accurate than open biopsy. Especially, in lipomatous tumors that are difficult to make a differential diagnosis by pathological findings, collecting sufficient samples by an accurate needle biopsy procedure is indispensable. Torriani et al. reported that sonographically guided procedures improved the diagnostic accuracy of needle biopsy for soft tissue tumors^38^. Therefore, in ALT/WDLS which are often developed in the deep site and showed irregular shapes, this technique will be helpful for accurate needle insertion and should be recommended.

Based on these results, in adipocytic tumors, the differential diagnosis should be evaluated based on a comprehensive assessment of clinical, radiological, and histopathological examinations. Although a few scoring systems for the differential diagnosis of ALT/WDLS based on radiological findings have been reported^1,7^, there is no diagnostic scoring system that includes histopathological findings. In the previous studies of similar scoring systems, Nagano et al. reported a sensitivity of 100% and specificity of 77%^1^, and Cheng et al. reported a sensitivity of 90% and specificity of 92.5%^7^. The diagnostic accuracy for ALT/WDLS of our scoring system is almost the same as in these studies. However, Nagano et al. study examined 48 lipomas and 12 ALTs, without including WDLS, and the case number was lower compared with our study^1^. In addition, this study investigated adipocytic tumors in all locations, but Cheng et al. investigated only deep-seated adipocytic tumors^7^. These limitations may have affected the difference in accuracy between our scoring system and those studies.

Although the diagnostic accuracy of our combined scoring system for ALT/WDLS was slightly lower than that of FISH for MDM2, our scoring system will help to preoperative differential diagnosis between ALT/WDLS and lipoma. Therefore, in the case that ALT/WDLS is suspected in this scoring system, sub-extensive resection will be recommended for surgeons. Furthermore, in our scoring system, if needle biopsy could not be performed, the AUC was 0.943 by the ROC curve and the diagnostic accuracy was high. The sensitivity and specificity were 94.4% and 80.4%, respectively, which is useful for screening test of ALT/WDLS in clinics where pathological examination cannot be performed. However, because of the risk of false-positives cases, if ALT/WDLS is suspected in this scoring system, consultation for specialists of oncology should be recommended.

This study had a limitation that the evaluation of specimens of needle biopsy may be affected by the biopsy site and the amount of sample. If a preoperative needle biopsy can be performed with an accurate procedure, the diagnostic accuracy for ALT/WDLS of this scoring system may be higher, which makes it a very useful diagnostic tool for the preoperative differential diagnosis of adipocytic tumors.

In conclusion, we developed a new combined scoring system based on a comprehensive assessment of clinical, radiological, and histopathological examinations for the preoperative differential diagnosis between ALT/WDLS and lipoma. This scoring system had high diagnostic accuracy for differential diagnosis of ALT/WDLS and was a useful preoperative diagnostic tool that anyone can use easily in many medical institutions.

## Materials and methods

### Study design and patients

This retrospective study included patients diagnosed with ALT/WDLS or lipoma from January 2005 to August 2021 collected from the database at Kanazawa University Hospital. A total of 337 patient charts were extracted, and 145 patients who underwent surgical resection after evaluation by MRI and needle biopsy were included as the development cohort in this study. The diagnoses were established by pathologists based on the resected specimens. Histologically, ALT/WDLS has been reported that the proliferation of mature and variably pleomorphic adipocytes containing single, enlarged, hyperchromatic nuclei, and intersection by fibrous septa are observed and distinguished from lipoma^2,17^. Of the 145 resected specimens, 82 cases were diagnosed with lipoma based on these histological features, but in the other 63 cases that differential diagnosis was histologically difficult were performed FISH of *MDM2* and/or *CDK4* and diagnosed. Spindle cell lipoma and pleomorphic lipoma which have relatively typical pathological findings, and tumor recurrences were excluded from this study. Furthermore, to assess the reproducibility of the combined scoring system for differential diagnosis between ALT/WDLS and lipoma, 40 patients with lipomatous tumors diagnosed between January 2020 and August 2021 were included in the validation cohort. The inclusion and exclusion criteria of the participants were the same as that of the development cohort, and evaluated the sensitivity, specificity, negative predictive value (NPV), positive predictive value (PPV), diagnostic accuracy, and kappa coefficient of the score as the cross-validation. This study was approved by the ethics committee of Kanazawa University Hospital, and Informed consent was obtained from all the participants. In addition, all methods were carried out in accordance with relevant guidelines and regulations.

### Data analyses

**Figure 4:**
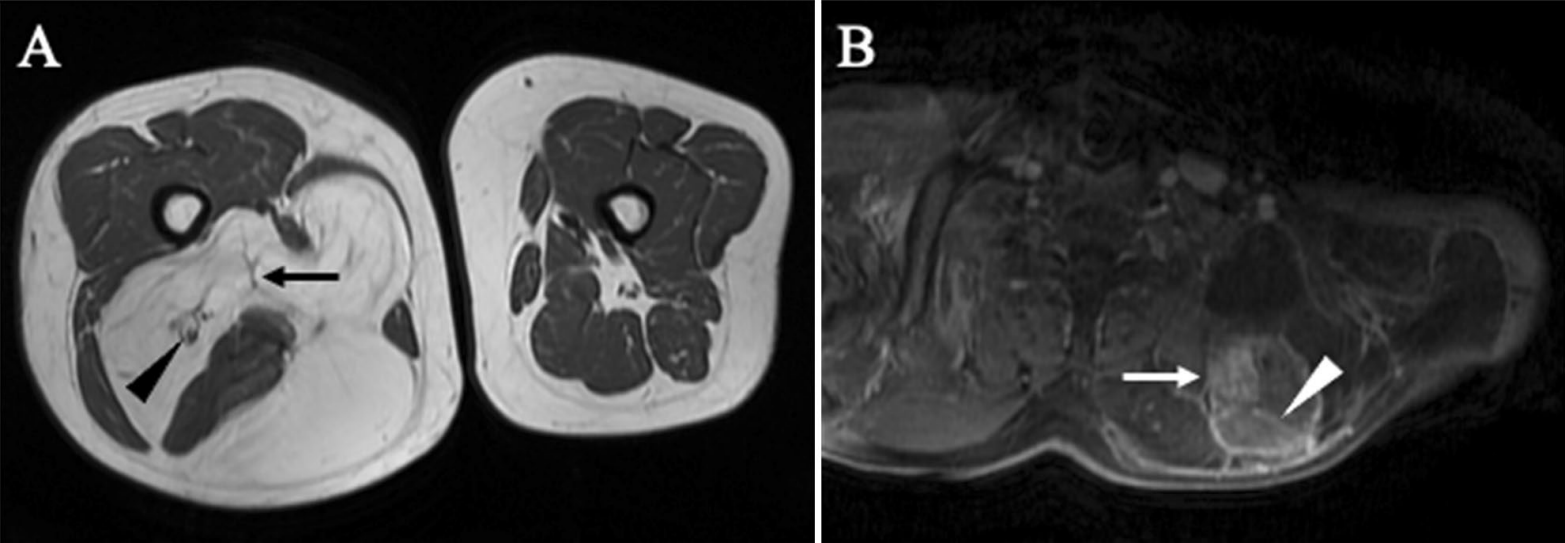
Radiological findings on MRI used in this study. (**A**) Thick septa (> 2 mm) (black arrow) and the sciatic nerve involved in the adipocytic tumor (black triangle) in T1-weighted image. (**B**). Enhancement of septa (white triangle) and nodular lesion (white arrow) in contrast-enhanced fat-suppression T2 image.

**Figure 5:**
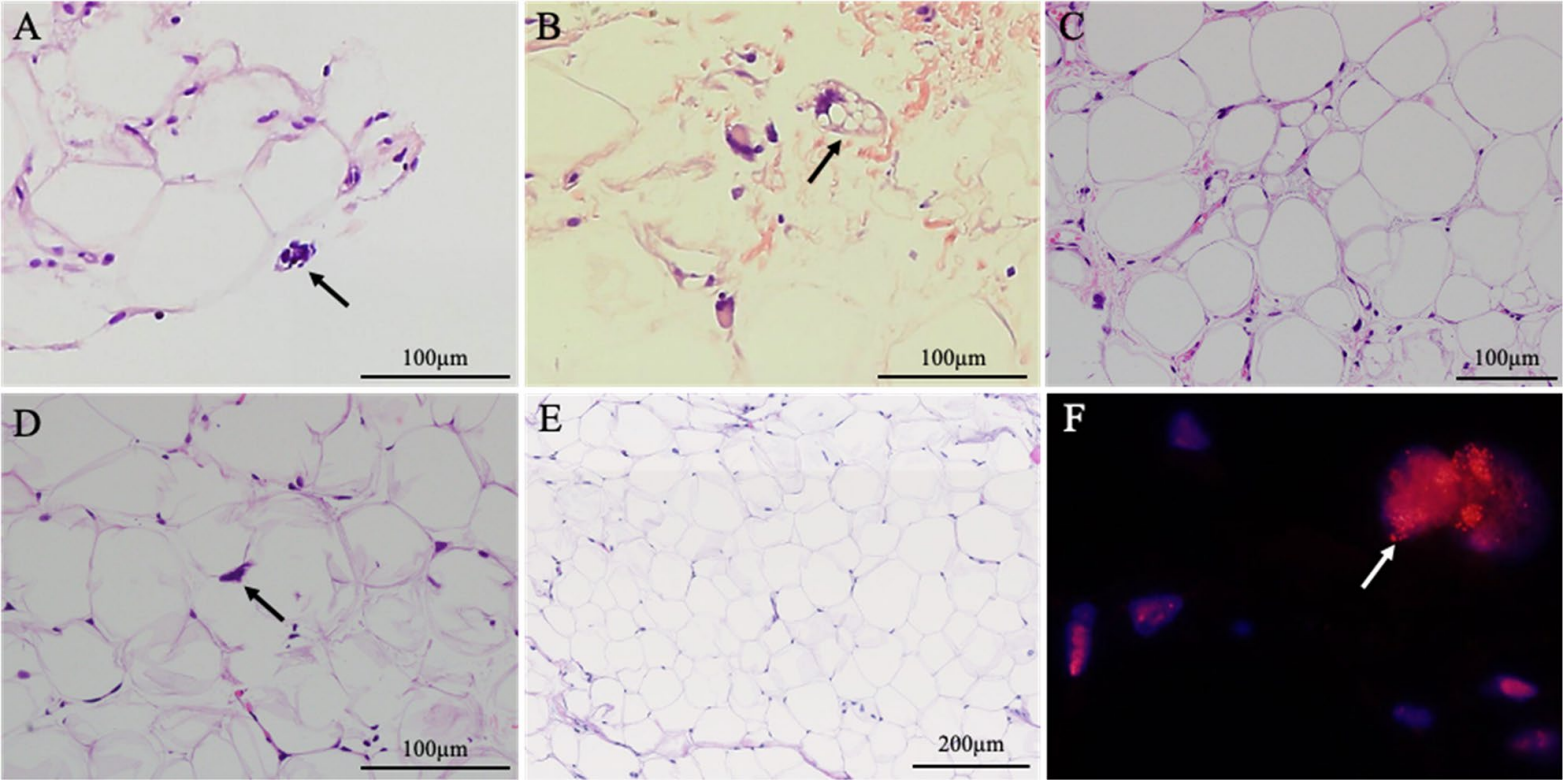
Histopathological findings by HE staining and FISH examination for MDM2 used in this study. (**A**) Nuclear atypia, (**B**) lipoblast, (**C**) differences in size of adipocytes and proliferation of fibrous septa, (**D**) nuclear enlargement, and (**E**) lipoma specimen occupied by mature adipocytes without the differences in size in HE staining. (**F**) MDM2 gene amplification was detected by FISH.

We collected the patients’ clinical characteristics, including age, sex, symptoms (pain and numbness), and tumor size, site, and depth from the medical records. The tumor size was measured by the greatest tumor diameter and the tumor depth was defined as a superficial or deep site with the fascia as the boundary by MRI (GE Healthcare Signa HDx 1.5 T, United States, GE Healthcare Signa HDxT 3.0 T, United States, and Philips Ingenia Elition 3.0 T, Netherlands). The MRI examination protocols were included sequences in sagittal, coronal, and axial planes using T1-weighted, T2-weighted, short T1 inversion recovery (STIR), and fat-suppressed T1 or T2-weighted images. Based on the previous studies regarding MRI findings used to distinguish ALT/WDLS and lipoma, radiologists evaluated three criteria: thick septa (> 2 mm), enhancement of septa or nodular lesions, and neurovascular involvement in the tumor^1,32,33^ (Fig. 4). The enhancement of septa or nodular lesions was evaluated on fat-suppressed T1 or T2-weighted images after the administration of contrast medium. All images were evaluated by 2 specialists of radiology, and in cases that their evaluations were equivocal, evaluated by another specialist and diagnosed with an agreement of inter-observer. Furthermore, based on histological findings by HE staining from needle biopsies specimens, the pathologists evaluated characteristics such as nuclear atypia and enlargement, proliferation of fibrous septa, differences in the size of adipocytes, and lipoblasts^2,8,33^ (Fig. 5). Lipoblasts were defined as the cells having hyperchromatic indented or sharply scalloped nuclei and lipid-rich mono- or multivacuolated cytoplasm^8,39^. In most patients diagnosed after 2013, the needle biopsy specimens were evaluated by FISH for *MDM2* and/or *CDK4* based on the recommended criteria for the test; deep lesions that are > 10 cm and in cases with equivocal atypia^11^. The probes used for the FISH analyses were Vysis LSI *MDM2* Spectrum Orange probe and Vysis CEP12(D12Z3) Spectrum Green probe (Abbott, USA) for *MDM2*, and Kreatech™ *CDK4*(12q13)/SE12 FISH probe (Leica Biosystems, Germany) for *CDK4*. FISH was performed using the same methods and protocols as previous report of our study group^10^.

### Statistical analyses

These characteristics and findings were compared between the ALT/WDLS and lipoma groups by Student’s t-test or the chi-square test. A p-value of less than 0.05 was considered statistically significant. Univariate logistic regression analysis was performed to determine the predictive factors for the diagnosis of ALT/WDLS and variables with p < 0.05 from univariate analysis were used for multivariate logistic regression analysis. A combined scoring system for the diagnosis of ALT/WDLS was developed according to the predictive factors that showed statistical significance by multivariate analyses. The accuracy of the diagnoses of the scoring system was evaluated using a ROC curve. All data analyses were performed using EZR^40^.
